# Addition of Honey to an Apple and Passion Fruit Mixed Beverage Improves Its Phenolic Compound Profile

**DOI:** 10.3390/foods10071525

**Published:** 2021-07-02

**Authors:** Iris Batista Leite, Carolina Duque Magalhães, Mariana Monteiro, Eliane Fialho

**Affiliations:** Functional Foods Laboratory, Nutrition Institute, Federal University of Rio de Janeiro, Av. Carlos Chagas Filho, 373 Bloco J, 2° Andar, Sala 16, Rio de Janeiro 21941-902, Brazil; irisbleite@gmail.com (I.B.L.); carolduquemagal@hotmail.com (C.D.M.); mariana@nutricao.ufrj.br (M.M.)

**Keywords:** hydroxycinnamic acids, antioxidant activity, multifloral honey, HPLC/MS

## Abstract

The addition of honey to mixed beverages is interesting due to its contribution to the sweet taste, as well as because it is a dietary source of bioactive compounds. In this study, we investigated the chemical composition and sensory acceptance of an apple and passion fruit mixed beverage with added honey. The addition of honey did not produce a noticeable change in instrumental color but led to an increase in total soluble solids contents, and FRAP (20%), TEAC (72%), and DPPH (62%) values. The honey mixed beverages exhibited a better phenolic compound profile with an increase in catechin contents and an enrichment of quercetin when compared to the control mixed beverage, as well presenting good sensory acceptance. In conclusion, our results show that the addition of honey can be an alternative for improving the nutritional and sensorial characteristics of an apple and passion fruit mixed beverage.

## 1. Introduction

Several epidemiological studies suggest that a balanced diet that includes the consumption of fruits and vegetables and their byproducts contributes to reducing the risk of developing non-communicable diseases, such as cardiovascular diseases, diabetes, and some types of cancers [1,2]. These preventive effects are partly associated with their high contents of bioactive compounds, which possess the capacity to influence either physiological or cellular activity and are beneficial to health [1]. In foods, these bioactive compounds are also responsible for sensorial attributes, such as color, astringency, and aroma, and improve their oxidative stability [3]. Among the bioactive compounds, the phenolic compound classes are notable for being widely found in different plant varieties, and their presence is essential for plant growth and reproduction, as they act as antipathogenic agents and contribute to food pigmentation [4].

One of the research team’s objectives was to compare the behavior of compounds that express a good synergistic effect in cancer cells and develop preparations that allow them to be consumed by the general population [5,6,7,8,9,10]. Therefore, the association of different food sources containing nutrients and bioactive compounds is interesting, and an apple and passion fruit mixed beverage with added honey serves this purpose. Moreover, passion fruit was used in this study as a source of ascorbic acid [11], which helps to preserve the oxidation of phenolic compounds present in apples during processing [12]. Apples are a fruit consumed worldwide and are rich in phenolic compounds such as quercetin and chlorogenic acids [13]. Additionally, several studies indicate the presence of phenolic compounds, such as quercetin-3-*O*-rutinoside and *p*-coumaric acid in passion fruits [14,15]. The addition of honey gives the drink a sweet taste and improved nutritional value due to the content of vitamins and minerals [16], and also due to its content of bioactive compounds, such as *p*-coumaric and caffeic acids [17], proving to be nutritionally superior to sugar and sweeteners [17,18].

Several varieties of honey are found, which are associated with the plant species from which bees collect their nectar, providing different chemical compositions and antioxidant activity [19]. Among the varieties of honey, the multifloral variety stands out for its high availability in the commercial market, since it comes from different types of flowers. Eucalyptus honey also has widespread commercialization in Brazil due to the reforestation of this tree, allowing for its flowers to be a source of raw material for bees. Due to its light color and its soft taste, orange blossom honey is also highly demanded by consumers. “Assa-peixe” honey is mainly produced in the southeast of Brazil and has a darker coloration and a characteristic flavor [20,21].

All three ingredients of the beverage have beneficial effects on health. Passion fruit consumption can have antihypertensive and lipidemic effects [22], apple consumption is able to prevent cancer and vascular disorders [23], and the addition of honey may bring health benefits such as improving the inflammatory state of obesity, and, more recently, has shown promising antiviral activity against pathogens that cause severe respiratory syndromes, as well as potential molecular action against coronavirus [24,25].

The present study was aimed at verifying the effect of the addition of honey to an apple and passion fruit mixed beverage on its instrumental color, total soluble solid contents, antioxidant activity, phenolic compound profile, and sensory acceptance.

## 2. Materials and Methods

### 2.1. Solvents, Reagents, and Standards

Potassium persulfate, sodium carbonate anhydrous, ferric chloride (FeCl_3_), 2,4,6-Tris(2-pyridyl)-*S*-triazine (TPTZ), 2,2′-azino-bis(3-ethylbenzothiazoline-6-sulphonic acid)diammonium salt (ABTS), 6-hydroxy-2,5,7,8-tetramethylchromane-2-carboxylic acid (Trolox), 2,2-Diphenyl-1-picrylhydrazy (DPPH), anhydrous sodium acetate, gallic acid, 5-caffeoylquinic acid, *p*-coumaric acid, quercetin, and catechin were purchased from Sigma-Aldrich Chemical Co. (St. Louis, MO, USA). Iron (II) sulphate was purchased from Merck (Darmstadt, Germany). Solvents (Tedia, Fairfield, OH, USA) and water (Milli-Q system, Millipore, Bedford, MA, USA) were of HPLC grade.

### 2.2. Samples and Mixed Beverage Processing

Passion fruits (*Passiflora edulis*, cv. Sims.) from the state of Santa Catarina, Brazil, were purchased during the harvest period (March 2016), at the Food Supply Centre of the State of Rio de Janeiro, Brazil. Fruits were selected and washed in distilled water, and the pulp was removed with a spoon. The passion fruit pulp was vacuum-packed in aseptic polyethylene bags and stored at −22 °C until use. Gala apples (*Malus domestica* cv. Borkh) were purchased from commercial establishments in the city of Rio de Janeiro, Brazil, washed in distilled water, and sanitized in 100 ppm sodium hypochlorite solution for 15 min. Both passion fruit and apple have similar harvest periods and were acquired covering the period of the best harvest. A total of 4 varieties of honey were purchased in a commercial establishment in the city of Rio de Janeiro in order to define which one would be added to the mixed beverage—eucalyptus (*Eucalyptus grandis*), multifloral, orange blossom (*Citrus spp*), and “assa-peixe” (*Vernonia brevifolia*).

The control mixed beverage (CMB) was prepared using 25 g of passion fruit pulp, 50 g of seedless gala apple, and 140 mL of filtered water. The honey mixed beverages (HMB) were prepared in the same manner as the CMB, with the addition of 9 g of honey [26,27]. In a blender, the ingredients were shaken for 3 min and then the juices were strained through a sieve, simulating a homemade beverage.

### 2.3. Instrumental Color Measurement and Total Soluble Solid Contents

The instrumental color parameters of the beverages (15 mL) were evaluated in triplicate using a Konica Minolta CR-400 colorimeter (Konica Minolta, Tokyo, Japan). The equipment was set to illuminant D_65_ (a 2° observer angle) and calibrated using a standard white reflector plate. The CIE-Labcolor space was used to determine the color components: L^*^(brightness or lightness; 0 = black, 100 = white), a* (−a*, greenness; +a*, redness) and b* (−b*, blueness; +b*, yellowness). The total color difference (ΔE*) between the CMB and the HMB was calculated using the following equation:

(1) $$\Delta E*=\sqrt{{\left(L_{CMB}^{*}-L_{HMB}^{*}\right)}^{2}+{\left(a_{CMB}^{*}-a_{HMB}^{*}\right)}^{2}+{\left(b_{CMB}^{*}-b_{HMB}^{*}\right)}^{2}}$$

where L*, a*, and b* were the color coordinates of the CMB and the HMB.

The total soluble solids (TSS) were measured using a portable refractometer (Model PAL-1; Atago, Tokyo, Japan). One drop of beverage was placed on the refractometer glass prism, and the TSS was obtained as °Brix.

### 2.4. Antioxidant Activity (AA)

The AA was determined in the beverages directly after centrifugation (11,300× *g*, 10 min) by FRAP (Ferric Reducing Antioxidant Power), TEAC (Trolox Equivalent Antioxidant Capacity), and DPPH (2,2-Diphenyl-1-picrylhydrazyl Free Radical) assays. Each sample was analyzed in triplicate.

The FRAP assay was performed with slight modifications based on the methodology described by Benzie and Strain (1996). FRAP reagent was prepared by mixing 2 mL of 10 mM TPTZ solution in 6 M HCl with 2 mL of 20 mM FeCl_3_ solution and 20 mL of 300 mM acetate buffer (pH 3.6) and warmed to 37 °C prior to analysis. Aliquots of juice (20 μL) were pipetted into a 96-well microplate and 180 μL of FRAP reagent was added. Microplate was placed in a spectrophotometer and then allowed to stand in the dark at 37 °C for 6 min. The absorbance was then read at 593 nm. Quantification was performed using a calibration curve prepared with FeSO_4_ and the results were expressed as mmol of Fe^2+^ equivalents per 100 mL [28].

The ABTS radical cation stock solution was generated by reacting K_2_S_2_O_8_ and ABTS for 12–16 h prior to use. The ABTS radical cation stock solution was diluted in water (1:50) to an absorbance of 0.70 ± 0.02 at 720 nm. Aliquots of juice (10 μL) were pipetted into a 96-well microplate and 190 μL of ABTS radical cation solution were added into each well, and the reagents were mixed by shaking and then allowed to stand at 37 °C for 6 min. The absorbance was read at 720 nm and subtracted from solvent blank absorbance. Quantification was performed using a Trolox calibration curve. Results were expressed as μmol of Trolox per 100 mL [29].

The DPPH assay was performed with slight modifications according to the method described by Duan et al. (2007). The 100 µM DPPH solution was prepared using 80% methanol. The absorbance of the DPPH solution in the absence of the sample was read in a spectrophotometer at 517 nm in order to verify the absorbance between 0.8 and 1.1. Then, 0.1 mL of each sample and 4.9 mL of the 100 µM DPPH solution were placed in test tubes. The tubes were manually shaken and kept in the dark for 30 min to take readings at 517 nm.

The percentage of DPPH radical inhibition was calculated with the following formula:

CA (%) = (A0 − A30)/A0 × 100(2)

where A0 represents the absorbance of the DPPH solution measured at the initial time and A30 is the absorbance of each sample 30 min after adding the DPPH solution. The results are expressed as the percentage of DPPH inhibition [30].

### 2.5. Phenolic Compound Profile by HPLC-DAD-MS

The beverages were analyzed directly after centrifugation (11,300× *g*, 10 min) and filtration through a 0.45 µm PTFE filter unit (Analitica^®^, São Paulo, Brazil). The liquid chromatography system (Shimadzu^®^, Kyoto, Japan) included two parallel LC-20AD pumps, a SIL-20AHT automatic injector, a CBM-20A system controller, a DGU-20A5 degasser, an SPD-M20A diode-array detector (DAD), and an LCMS-2020 quadrupole mass spectrometer with electrospray ionization (ESI). A N_2_ generator (NM32LA, Peak Scientific^®^, Inchinnan, Renfrewshire) was coupled to the LCMS.

Chromatographic separation of phenolic compounds was achieved using a reverse-phase column (phenyl, 5 μm, 250 mm × 4.6 mm, Phenomenex^®^, Torrance, Canada). The mobile phase consisted of a gradient of 0.3% formic acid and 1% acetonitrile in water (eluent A) and 1% acetonitrile in methanol (eluent B), with a flow rate of 1.0 mL/min. Prior to the injection, the column was balanced with 18.2% B. After the sample injection, the solvent composition changed to 20.2% B in 1 min, to 43.4% B in 18 min, 85.9% B in 23 min, and kept constant until 30 min. Between injections, 10 min intervals were used to rebalance the column with 18.2% B. The phenolic compounds were monitored from 190 to 530 nm [31].

The ESI interface was operated in selected ion monitoring (SIM) and SCAN negative mode for phenolic compound ionization. The MS operation conditions were as follows: detector voltage of 3.0 kV; interface temperature of 350 °C; desolvation line temperature of 250 °C; nebulizing gas (ultra-pure N_2_) flow of 1.5 L/min; heat block of 200 °C; drying gas (ultra-pure N_2_) flow of 15 L/min.

5-Caffeoylquinic acid, 4-caffeoylquinic acid, quercetin-3-*O*-glucoside, catechin, and quercetin were identified by comparison with the retention time, absorption spectra, and *m/z* of the ions of the respective commercial standard. Quantification was performed by external standardization using the DAD signal. The LOD and LOQ for phenolic compounds were equal to or lower than 0.006 mg/100 mL and 0.017 mg/100 mL. *p*-Coumaroylquinic acid isomers were tentatively identified by their pseudo-molecular ions and quantified using commercial standards of *p*-coumaric acid. Integration data were acquired by the Lab Solutions software (Shimadzu Corporation^®^, Kyoto, Japan, version 5.82 SP1, 2008–2015).

### 2.6. Sensory Analysis

Sensory acceptance and purchase intent were carried out with a group of 101 untrained consumers (83 females and 18 males) aged between 17 and 63 years. The subjects were recruited at the Federal University of Rio de Janeiro, Brazil, and included staff, students, and visitors. Only the HMB prepared with multifloral honey was chosen to perform the sensorial tests, as this honey presented good results in previous analyses, as well as being more commercialized and cheaper [20].

A sample (25 mL) of HMB was offered at ~10 °C in 50 mL plastic cups coded with three-digit numbers and offered monadically in balanced order [32]. The following acceptance attributes were evaluated using a 9-point structured hedonic scale (1 = I extremely dislike; 9 = I extremely like): overall impression, aroma, taste, and color. The purchase intent was evaluated by a 5-point structured hedonic scale (1 = I would definitely not buy it; 5 = I would definitely buy it) [33]. Sensory analyses were conducted in a laboratory free from noise and odor and with individual benches to conduct the sensory analysis with minimal distractions. The study was approved by the ethics committee of the Federal University of Rio de Janeiro (approval number: 1,906,704). Before participating in the study, the subjects were asked to sign an informed consent form.

### 2.7. Statistical Analysis

The data are expressed as the mean ± standard deviation. The effect of the addition of honey on all attributes was evaluated by one-way ANOVA, followed by Tukey’s multiple comparisons posthoc test using the GraphPad Prism software for Windows (version 5.01, GraphPad Software, San Diego, CA, USA). The results are considered significant when *p* < 0.05.

## 3. Results and Discussion

### 3.1. The Addition of Honey Increases Total Soluble Solid Contents and Does Not Change Perception of Color of Mixed Beverages

The addition of honey led to an average increase of 87% in TSS (Table 1), which is attributed to the presence of sugars (glucose and fructose) in honey, which account for 85% of its composition [34]. TSS in the CMB also was lower than those reported for mixed passion fruit juice (11.7 °Brix) and apple juices (10.4 °Brix to 14.9 °Brix) [35,36], probably due to the addition of water that was used to prepare the CMB, which dilutes soluble solids [37].

The luminosity (L*) of the HMB (38.4, on average) was lower than that observed for the CMB (40.1) (Table 1), indicating that the addition of honey made the mixed beverages slightly darker. The a* values (−0.44, on average) and b* values (18.42, on average), were respectively higher and lower than those observed for the CMB (−0.52 and 19.51), indicating that these beverages were slightly reddish and bluish. The ΔE* values, which correspond to the total color difference between the CMB and HMB were, on average, 2.04, indicating that the instrumental color differences due to the addition of honey would be hardly distinguishable by the human eye.

### 3.2. The Addition of Honey Increases Antioxidant Activity and Improves Phenolic Compound Profile of Mixed Beverages

The AA of the CMB was 109.7 mmol Fe^+2^/100 mL, 38.8 μmol Trolox/100 mL, and 5.3% of DPPH radical, as evaluated by the FRAP, TEAC, and DPPH assays, respectively (Table 2).

In general, the addition of honey led to an increase of 20%, 72%, and 62% in AA, as evaluated by the FRAP, TEAC, and DPPH assays, respectively. Comparing all the HMB, the beverage with the added eucalyptus honey showed the lowest AA, as evaluated by all assays. In accordance with our study, other authors reported an increase in the DPPH values of teas [22] and infusions [38] with added honey. Increases in the FRAP values of infusions with added honey and in the TEAC values of tea with added honey are also reported in the literature [39].

**Figure 1:**
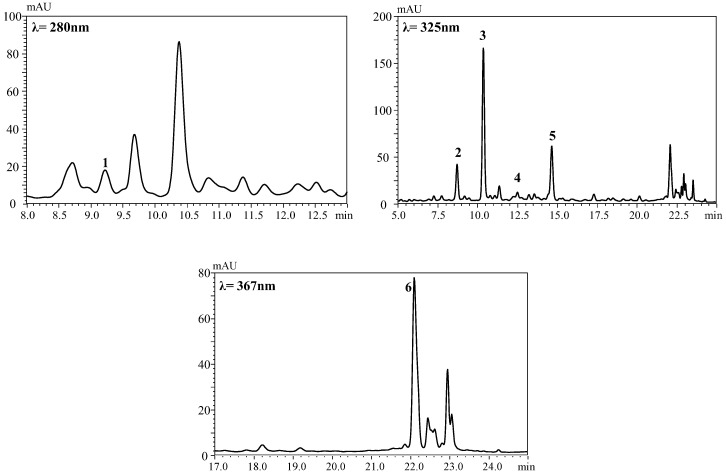
HPLC-DAD chromatograms of the phenolic compounds of the control mixed beverage. The compounds identified were catechin (1), 4-caffeoylquinic acid (2), 5-caffeoylquinic acid (3), *p*-coumaroylquinic acid isomer 1 (4), *p*-coumaroylquinic acid isomer 2 (5), and quercetin-3-*O*-glucoside (6).

A total of 6 phenolic compounds were identified in the CMB, 4 of which were hydroxycinnamic acids derivatives (5-caffeoylquinic acid, 4-caffeoylquinic acid, and 2 isomers of *p*-coumaroylquinic acid), 1 flavonol (quercetin-3-*O*-glucoside), and 1 flavanoid (catechin) (Figure 1).

**Figure 2:**
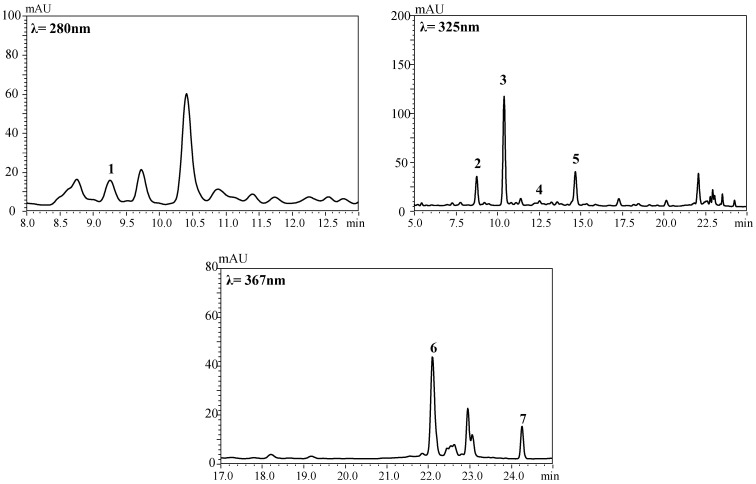
HPLC-DAD chromatograms of the phenolic compounds of the honey mixed beverage. The compounds identified were catechin (1), 4-caffeoylquinic acid (2), 5-caffeoylquinic acid (3), *p*-coumaroylquinic acid isomer 1 (4), *p*-coumaroylquinic acid isomer 2 (5), quercetin-3-*O*-glucoside (6), and quercetin (7).

**Figure 3:**
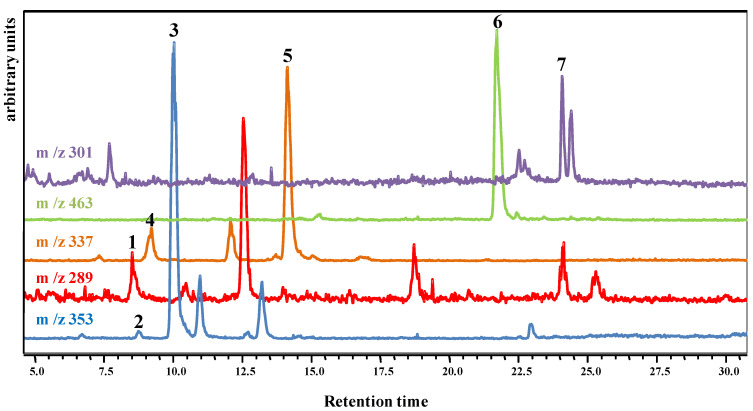
HPLC-MS chromatogram of the phenolic compounds of the honey mixed beverage. The compounds identified were catechin (1; *m*/*z* = 289), 4-caffeoylquinic acid (2; *m*/*z* = 353), 5-caffeoylquinic acid (3; *m*/*z* = 353), *p*-coumaroylquinic acid isomer 1 (4; *m*/*z* = 337), *p*-coumaroylquinic acid isomer 2 (5; *m*/*z* = 337), quercetin-3-*O*-glucoside (6; *m*/*z* = 463), and quercetin (7; *m*/*z* = 301).

The HMB presented all the phenolic compounds identified in the CMB in addition to quercetin (Figure 2 and Figure 3). This phenolic compound profile was in accordance with previously published data for passion fruit and apple fruits and honey. Among them, 5-caffeoylquinic acid, 4-caffeoylquinic acid, and *p*-coumaroylquinic acids have been reported in apple [40], quercetin-3-*O*-glucoside in passion fruit and apple [41,42], and quercetin and catechin in honey [43,44] Other phenolic compounds have already been described in the ingredients presented, especially honey [45]. A limitation may refer to the method used to extract and quantify polyphenols. Although we have used a methodology already well described in our field and published with the use of different food matrices, the quantification may not express the real phenolic amounts in the mixed beverage.

In addition to the fact that quercetin has already been reported in honey, its presence in the HMB, related to the concomitant reduction of quercetin-3-*O*-glucoside, may also be explained by the presence of β-glucosidase in honey [46]. This enzyme is capable of promoting the deglycosylation of glycosylated forms of quercetin to aglycone ones [47].

In Table 3, the decrease in the averages of the honey mixed beverages of 5-caffeoylquinic acid, 4-caffeoylquinic acid, *p*-coumaroylquinic acid isomers 1 and 2 can be observed. The decrease of these compounds may be due to the possible presence of Aspergillus niger, found in many varieties of honey [48]. This non-pathogenic microorganism is commonly used in the food industry, and the intrinsic activity of this chlorogenic acid hydrolase activity can hydrolyze the methyl ester of caffeic and *p*-coumaric acids [49,50,51,52]. In fact, Asther et al. (2005) demonstrated the effect of the purified enzyme of this fungus on apple pomace and observed the hydrolysis of more than 80% of chlorogenic acid [51].

Additionally, the levels of catechin were enhanced more than 2.5 times and quercetin was present only in the HMB. These modifications are associated with the increase in AA in the HMB based on different profiles of phenolic compounds. In fact, the AA of foods depends on the phenolic compound chemical structure and varies according to their glycosylation and the position and number of hydroxyl groups [53,54,55]. The following TEAC values of phenolic compounds isolated from Chinese medicinal plants can be found in the literature: 5-caffeoylquinic acid (1.56 mM of Trolox equivalents—TE), quercetin-3-*O*-glucoside (2.39 mM TE), catechin (3.04 mM TE), and quercetin (4.42 mM TE) [54]. These values are similar to those reported in another work (1.24 mM TE for 5-caffeoylquinic acid, 2.4 mM TE for catechin, and 4.7 mM TE for quercetin), which reinforces the influence of the chemical structure of bioactive compounds on AA [55].

In the present study, 5-caffeoylquinic acid and quercetin-3-*O*-glucoside, compounds reported in the literature to have lower AA, contributed to 69% of the total phenolic content in the CMB and, on average, to 52% in the HMB. On the other hand, catechin, which had an intermediary TEAC value but was higher than the previous phenolic compounds, represented 8.3% of total phenolics in the CMB and, on average, 23.3% in the HMB. Finally, quercetin, which had a higher TEAC value than the other phenolic compounds, was only identified in the HMB, representing, on average, 6.9% of the total phenolic contents in the HMB.

Other aspects of honey that can be taken into account are the micronutrients that can be accumulated in the beverage, such as zinc, calcium, ascorbic acid, and some complex B vitamins. This ingredient can also bring carbohydrates and calories to the beverage, but the amount of honey used in this beverage represents an increase of no more than 29 kcal [55]. The presence of more phenolic compounds in the HMB and the possibility of the synergistic effect of phenolic compounds identified is more representative [4,56,57].

In this context, the use of honey to sweeten this beverage is interesting because it does not provide a significant amount of calories and is capable of providing different nutrients and bioactive compounds for the consumer.

### 3.3. Sensory Acceptance Analysis

Consumers accepted the HMB well, and the attributes of aroma and appearance received the highest scores (8.02 ± 1.09 and 8.42 ± 0.77, respectively), and corresponded to the parameters between “I moderately like” and “I extremely like”. The addition of honey to the apple and passion fruit beverage did not negatively influence the color attribute and, in relation to the aroma, the attribute was within the acceptability range [58]. Overall impression (7.76 ± 0.91) and taste (7.53 ± 1.22) obtained scores corresponding to “I regularly like” and “I moderately like”. The purchase intent of the HMB product (4.01 ± 0.71) obtained scores corresponding to “I would probably buy”, indicating that most of the people would buy this beverage if it were marketed.

From the consumers’ comments, it can be inferred that the lowest score of the HMB taste is related to the fact that they expected the most notable taste from the passion fruit. Additionally, consumers reported that the HMB had a soft taste, which can be explained by the water dilution and the addition of apple. The questionnaire administered before the sensory acceptance analysis included four questions related to the consumption of the mixed beverages and honey, and when people were asked if they consumed any type of mixed beverage (more than one ingredient), 39.6% of the consumers reported that their consumption was moderate, and most of them indicated a frequency of two times a month. Regarding the frequency of honey consumption, 37.6% of the consumers reported that it is occasional, with the majority saying five times a year. Among the consumers that reported honey consumption, 33.3% related their use in tea and 30% in fruit juices.

As can be seen from the above data, consumers reported a low frequency of honey consumption. In 2014, a Brazilian study reported an average honey consumption of 49 g per capita per year, which is much lower than those reported in other countries, such as the United States (910 g per year) and Switzerland (1500 g per year) [59]. Most honey consumption is in the usual way since the majority of the population considers it a natural medicine [60]. The use of honey in beverages would be a way to increase its consumption. Positive results for the purchase intent of cashew nectar sweetened with honey were reported [58]. The most frequent scores were “maybe I would buy it, maybe I would not buy it” and “I would probably buy it”, which is similar to the one found in the present study where most evaluators indicated that they would “possibly yes” buy the product.

## 4. Conclusions

Based on the results obtained, honey mixed beverages containing orange blossom, multifloral, and “assa-peixe” honey presented the highest antioxidant activity values measured by FRAP, TEAC, and DPPH assays. The beverages containing eucalyptus and orange blossom honey showed a higher content of total phenolic compounds compared to the other kinds of honey and similar to the control beverage, however, with a different phenolic profile. This difference was marked with the increase of catechin and the presence of quercetin in beverages with added honey, with expressive antioxidant compounds. These results suggest that the addition of honey to mixed beverages is favorable to improve their quality since they did not change their instrumental color, and they improved their phenolic compounds profile, enhanced their antioxidant activities, and presented a good sensorial acceptance. Therefore, these kinds of honey would be good options to add nutritional value to a mixed beverage based on apple and passion fruits.

## Figures and Tables

**Table 1:** Total soluble solids (TSS) and instrumental color of the control mixed beverage and the honey mixed beverages.

Component	Control Mixed Beverage	Honey Mixed Beverages			
		Orange Blossom	Eucalyptus	Multifloral	Assa-Peixe
TSS (°Brix)	4.33 ± 0.03 ^c^	7.93 ± 0.03 ^b^	8.20 ± 0.00 ^a^	8.03 ± 0.03 ^b^	7.93 ± 0.03 ^b^
L *	40.10 ± 0.00 ^a^	38.26 ± 0.01 ^e^	38.43 ± 0.01 ^c^	38.48 ± 0.01 ^b^	38.37 ± 0.01 ^d^
a *	−0.52 ± 0.01 ^a^	−0.37 ± 0.006 ^b^	−0.49 ± 0,01 ^a^	−0.56 ± 0.01 ^a^	−0.34 ± 0.02 ^b^
b *	19.51 ± 0.01 ^a^	18.35 ± 0.01 ^c^	18.38 ± 0.02 ^c^	18.55 ± 0.01 ^b^	18.40 ± 0.01 ^c^

Results are expressed as mean ± standard deviations of three replicates. Different superscript letters in the same row indicate significant differences between mean values (one-way ANOVA followed by Tukey multiple comparisons posthoc test, *p* < 0.05). The CIELab color space was used to determine the coordinates *L* * (black (0) to white (100)), *a* * (green (−) to red (+)), and *b* * (blue (−) to yellow (+)).

**Table 2:** Antioxidant activity of the control mixed beverage and the honey mixed beverages, measured by FRAP, DPPH, and TEAC assays.

Method	Control Mixed Beverage	Honey Mixed Beverages			
		Orange Blossom	Eucalyptus	Multifloral	Assa-Peixe
FRAP (mmol Fe^+2^/100 mL)	109.7 ± 0.3 ^b^	140.8 ± 1.1 ^a^	97.9 ± 2.8 ^c^	149.9 ± 3.5 ^a^	139.1 ± 3.2 ^a^
TEAC (μmol Trolox/100 mL)	38.8 ± 0.3 ^c^	70.4 ± 1.6 ^a^	50.4 ± 2.1 ^b^	71.3 ± 1.6 ^a^	74.7 ± 1.3 ^a^
DPPH (% of DPPH radical inhibition)	5.3 ± 0.3 ^b^	9.4 ± 0.7 ^a^	6.8 ± 0.4 ^a,b^	10.1 ± 1.4 ^a^	8.2 ± 0.5 ^a,b^

Results are expressed as mean ± standard deviations of three replicates. Different superscript letters in the same row indicate significant differences between mean values (one-way ANOVA followed by Tukey multiple comparisons posthoc test, *p* < 0.05).

**Table 3:** Phenolic compound contents (mg/100 mL) of the control mixed beverage and the honey mixed beverages.

Phenolic Compound	Control Mixed Beverage	Honey Mixed Beverage			
		Orange Blossom	Eucalyptus	Multifloral	Assa-Peixe
5-Caffeoylquinic acid	9.54 ± 0.09 ^a^	6.90 ± 0.22 ^c^	7.65 ± 0.01 ^b^	7.06 ± 0.16 ^c^	6.63 ± 0.09 ^c^
4-Caffeoylquinic acid	0.79 ± 0.03 ^a^	0.67 ± 0.03 ^a^	0.81 ± 0.03 ^a^	0.79 ± 0.05 ^a^	0.53 ± 0.03 ^b^
*p*-Coumaroylquinic acid isomer 2	2.82 ± 0.08 ^a^	1.95 ± 0.01 ^b^	2.09 ± 0.06 ^b^	1.97 ± 0.17 ^b^	1.90 ± 0.03 ^b^
*p*-Coumaroylquinic acid isomer 1	0.64 ± 0.04 ^a^	0.23 ± 0.01 ^b^	0.41 ± 0.06 ^b^	0.23 ± 0.03 ^b^	0.35 ± 0.07 ^b^
Quercetin-3-*O*-glucoside	3.24 ± 0.03 ^a^	1.86 ± 0.01 ^b^	1.79 ± 0.01 ^b,c^	1.71 ± 0.02 ^c^	1.74 ± 0.01 ^c^
Catechin	1.53 ± 0.16 ^b^	4.26 ± 0.27 ^a^	4.03 ± 0.27 ^a^	3.95 ± 0.06 ^a^	3.71 ± 0.18 ^a^
Quercetin	ND	1.10 ± 0.01 ^b^	1.40 ± 0.04 ^a^	1.09 ± 0.02 ^b^	1.14 ± 0.03 ^b^

Results are expressed as mean ± standard deviations of three replicates. Different superscript letters in the same row indicate significant differences between mean values (one-way ANOVA followed by Tukey multiple comparisons posthoc test, *p* < 0.05). ND = not detected.
